# Retinal tear formation after whole-body vibration training exercise

**DOI:** 10.1186/s12886-019-1291-y

**Published:** 2020-01-29

**Authors:** John Maggiano, Mei-Chuan Margret Yu, Sanford Chen, Timothy You, Rajiv Rathod

**Affiliations:** Orange County Retina Medical Group, 1200 North Tustin Ave, Suite 140, Santa Ana, Santa Ana, CA 92705 USA

**Keywords:** Retinal tear, Vitreous hemorrhage, Whole-body vibration training

## Abstract

**Background:**

The purpose of this report is to describe the occurrence of a retinal tear, pre-retinal and vitreous hemorrhages after completing a session of whole-body vibration training. Though our patient is the third documented case of vitreous hemorrhage following whole-body vibration training, he is the first to show a close association of a retinal tear and vitreous hemorrhage with whole-body vibration training.

**Case presentation:**

This case report describes a 59-year-old male who had acute onset of floaters and shadows in the left eye. The results of the examination revealed a temporal retinal tear, inferior pre-retinal hemorrhage, and mild vitreous hemorrhage. He was successfully treated using laser photocoagulation with resolution of vitreous and pre-retinal hemorrhages on 4-month follow up.

**Conclusions:**

With the rise in popularity of whole-body vibration training exercise, it is important for the medical and athletic community to be aware of possible associated ocular complications. We believe that high-force vibration training may cause retinal tears in susceptible persons.

## Background

Retinal tears may occur when the vitreous exerts traction on the retinal tissue. Often, vitreous hemorrhages may simultaneously occur during this process. We present a case report of a patient who developed both a retinal tear and vitreous hemorrhage soon after whole-body vibration training. In current literature, there have only been two documented cases of vitreous hemorrhage following whole-body vibration exercise [1, 2]. Both of these papers suggest a high probability of correlation between whole-body vibration training and subsequent vitreous hemorrhage.

## Case presentation

A 59-year-old male presented with a chief complaint of floaters and a moving shadow in the left eye within a few hours of completing a session of whole-body vibration training. He reported using the machine three times that first and only day of use, for 60 s each time, thirty minutes apart. His feet were positioned 12 in. apart while wearing tennis shoes. Each time there was a full isometric effort with stiffening of his entire body (legs, back, abdomen, chest, and arms). Approximately 6 h after using the machine that same day, he developed his first symptoms: “spots” in vision, then “curtain-like things and strings” and were progressive over the next 3 days. On the fourth day, retinal examination revealed a temporal retinal tear, mild vitreous hemorrhage, and an inferior pre-retinal hemorrhage in the left eye. His vision was 20/30 in the right eye with spectacle correction and 20/30^− 2^ in the left eye with spectacle correction. Laser photocoagulation of the tear was successfully completed that same day. The patient had a previous history of bilateral radial keratotomy in both eyes more than 20 years prior to this incident. Prior to radial keratotomy his prescription was − 6.50 diopters sphere in the right eye and − 7.00 diopters sphere in the left eye, with both eyes correctable to 20/20. His health history was positive for hyperlipidemia, hypertension, and adult onset diabetes mellitus without ocular manifestations. No other significant family or psychosocial history was reported. At the patient’s 4-month follow up there was resolution of the vitreous and pre-retinal hemorrhage without new tears seen. His vision at the 4-month follow up was pinhole 20/25^− 2^ in both eyes.

## Discussion and conclusions

High level whole-body vibration training induces isometric muscle exercise in many muscles throughout the body, especially when in the standing position. Potential benefits include use in physical therapy and sports training, and such exercise is generally thought to increase bone and muscle health [3]. One case study described a reduction in diabetic peripheral neuropathic pain with use of whole-body vibration therapy [4].

Vibration and shaking have been shown to have effects on various tissues in the human body [3]. Vibration can be caused by situations such as occupational environment (e.g. pneumatic drill operation), trauma (e.g. shaken baby syndrome), and whole-body vibration training exercise. Some documented findings of adverse vibration after-effects include vasospastic diseases in hands [5] as well as ocular effects such as increased pigment within the trabecular meshwork, vitreous liquefaction [6] and retinal and vitreous bleeding in child abuse [7]. Other harmful ocular effects from vibratory sources include sling-shot forces and bungee cord jumping, which may produce an exaggerated vitreo-retinal traction effect.

Our patient had utilized a whole-body vibration training device for 60 s three times a few hours before the onset of his ocular symptoms. He reported that his head shook and teeth chattered during the exercise and that the forces to his body were quite severe. He was in a standing position holding the handlebars with his knees straight. The device used by our patient included a footplate on which the user can position him or herself that vibrates at a frequency up to 25–50 Hz. One study details how mechanical resonant frequency can affect the eye, outlining different vibration frequencies between 5 to 50 Hz with secondary transmission frequency to the eye [8]. This study suggests the approximate resonant frequency of the eye to be 18 Hz and a partial resonance of 10–12 Hz for the vitreous body [8].

In the case of our patient, it is hypothesized that one possible pathogenesis of the retinal tear and vitreous hemorrhage was instigated by the vibrations from the training machine transmitting a vibratory force to the eye and vitreous. In various types of vitreous-induced retinal tear formation, it is thought that vitreous movement causes shear injury to the retina at the vitreous base. The tear in our patient occurred at the vitreous base. The vibratory force probably caused his retinal tear and vitreous hemorrhage since the onset of symptoms was within 24 h of using the whole-body vibration training device. We believe our patient and those in the Bertschinger and Gillan papers strongly suggest that high-force vibration training may cause retinal tears in susceptible persons. A vitreous hemorrhage without retinal tears was reported in a 43-year-old high-myope by Bertschinger; this occurred within 2 weeks after beginning twenty-minute sessions twice per week. Gillan reported a case of a 52-year-old man who had previously used whole-body vibration training without symptoms but after a single 20-min session of training he developed a new floater within 2 min of completion and a significant drop in vision within 5 min. Though our patient is the third documented case of vitreous hemorrhage following whole-body vibration training, he is the first to show probable causation of a retinal tear and vitreous hemorrhage with whole-body vibration training.

## Data Availability

Not applicable. Data sharing is not applicable to this article as no datasets were generated or analyzed during the current study.
